# Facilitators and barriers in preventing doping among recreational athletes: A qualitative interview study among police officers

**DOI:** 10.3389/fpubh.2022.1017801

**Published:** 2022-10-05

**Authors:** Pia Kvillemo, Anna K. Strandberg, Tobias H. Elgán, Johanna Gripenberg

**Affiliations:** ^1^Department of Clinical Neuroscience, Stockholm Prevents Alcohol and Drug Problems (STAD), Centre for Psychiatry Research, Karolinska Institutet, Stockholm, Sweden; ^2^Stockholm Health Care Services, Stockholm, Sweden

**Keywords:** anabolic androgenic steroids, performance and image enhancing substances, public health, policy, control, recreational sport, gym, multi-component program

## Abstract

**Background:**

Doping is a societal problem associated with health problems, violence, and other crimes, especially when combined with alcohol and drugs. Elite, as well as recreational athletes who exercise in gyms may use doping to enhance their performance and/or improve their appearance. According to Swedish law, manufacturing, selling, supplying, possessing, and using anabolic androgenic steroids and growth hormones is forbidden. Exceptions apply if these substances are used for medical purposes and prescribed by doctors. As doping is illegal, the police authority is vital in counteracting doping.

**Aim:**

We aimed to identify facilitators and barriers to effective doping prevention at gyms by examining police officers' views on doping as a societal problem, their experiences of doping prevention efforts, and their perceptions on what enables or hinders doping prevention.

**Methods:**

Interviews with police officers (*n* = 15) were conducted from December 2021 to May 2022. The interviews were recorded and transcribed verbatim. A targeted content analysis of the material was performed.

**Results:**

Facilitators for effective doping prevention involving the police included the recognition of doping as a societal problem; mobilization of key actors; motivated police management and officers; adequate resource allocation; collaboration between the police, gyms, and other relevant authorities; and skills development for police and other professions. Barriers to effective doping prevention included a lack of knowledge about doping, time-consuming processes around the detection and collection of evidence in doping offenses, and competing tasks for police officers.

**Conclusion:**

Doping prevention should become more efficient by taking advantage of existing facilitators and removing remaining barriers. This study could guide recommendations linked to the police organization and the surrounding society regarding doping prevention.

## Introduction

In recent decades, doping has attracted attention as a growing societal problem (1–5). Elite athletes, as well as recreational athletes who exercise in gyms use doping to enhance their performance and/or improve their appearance (6, 7). In 1991, Sweden implemented its first doping law (8), which banned the manufacture, sale, supply, possession, and use of anabolic androgenic steroids and growth hormones. Exceptions applied if the substances were used for medical purposes and prescribed by physicians. The law was implemented as the use of hormone preparations for doping purposes posed significant public health problems (5).

Examples of mental and physical consequences of doping include mood swings, aggression, decreased empathy, depression, infertility, liver damage, muscle damage, and cardiac injuries, such as fibrosis, cardiac hypertrophy, and dilated cardiomyopathy with an increased risk for myocardial infarction, arrhythmias, and sudden cardiac death. Common mechanisms in the damaging process are oxidative stress, apoptosis, and protein synthesis alteration, involving primarily the cardiovascular system and the reproductive system. The use of doping before the body is fully developed can also cause premature puberty and a shortened body length. In addition to individual health problems and premature death, doping is also associated with violence against other people and other crimes, especially when combined with alcohol and drugs (9–18). Related to these problems, Zaami et al. found common reports of linkages between a number of psychopathological disorders and consumption of appearance and performance enhancing drugs, when reviewing the literature on the effects of such drugs on personality traits (18).

In Sweden, a formalized difference is made between the use of prohibited performance-enhancing agents within elite sports and recreational sports. Recreational sports are regulated by the Swedish doping law, while elite sports are also regulated by the regulations set by the World Anti-Doping Agency. In order to ensure that doping laws and regulations are complied with, controls are conducted by the organization Antidoping Sweden. According to the organization's own statistics, about 3,000–4,000 controls are performed annually, whereof 85% of these are done with elite athletes and the remaining 15% with lower-level competitive and recreational sports. In addition, the Police Authority is a key stakeholder as they are to ensure that the Swedish doping law is complied with within for example recreational sports at training facilities.

The number of people using doping substances in Sweden is difficult to estimate, but several recurring surveys have asked whether the responders participate in doping. In the 2019 school survey among students in the ninth grade and second grade in upper secondary school (tenth grade), approximately 1–2% of the boys and 0–1% of the girls reported use of anabolic androgenic steroids (19). The most recent Swedish national public health survey also showed that 1% of men and <0.5% of women aged 17–84 reported a lifetime use of anabolic androgenic steroids or growth hormones (20). Very few reported having used such substances in the past month. However, studies with self-reported data from the Nordic countries indicate a potential increase in doping during the last decades (3). Moreover, a recent survey in Sweden showed that the lifetime prevalence of the use of performance and image enhancing substances was approximately 12% among young people aged 16–25 (5).

The growing concern about doping occurring outside the organized sporting system resulted in the European Union (EU) Commission producing a review on the evidence base for policies to combat doping in recreational sport in 2014 (21). Doping prevention in recreational sports relies primarily on education and information interventions along with legislative measures and control. The authors of the EU Commission report concluded that published studies examining the effects of anti-doping education programmes were rare, however some experts contributing to the literature review claimed, based on previous studies, that educational media campaigns could have intended effect. Some support for combined educational programmes and practical strength training, targeting adolescents and students, was obtained later in a single study from 2016 (22) and it has also been recommended that prevention (educational) efforts should involve key stakeholder groups, such as coaches (23).

Given the fact that most legal, administrative, and political arrangements regarding doping prevention in relation to recreational sports were fairly recent in 2015, the EU Commission report could not present consensus on what a good practice might look like with respect to these factors (21). Some years later in 2021, Bates and Vinther conducted a literature review on doping prevention interventions, concluding that the evidence base remained underdeveloped and effects still unclear (4), highlighting the importance of implementation and intervention science to improve the quantity and quality of the evidence base for doping prevention efforts. The authors also emphasized the importance of supporting the development of interventions that are appropriate, motivated, feasible, sustainable, and consistent with the needs of those to whom they are addressed.

During the 1990s, gyms in different parts of Sweden experienced problems with doping at certain training facilities used primarily by recreational athletes. This led to a series of initiatives to prevent the harmful consequences of doping around the country. STAD (Stockholm Prevents Alcohol and Drug Problems) applied for funding from the Swedish Public Health Agency in 2007 to develop, implement, and evaluate a method for preventing doping in recreational sports in co-production (cooperation) with relevant actors. The method, called 100% Pure Hard Training (100% PHT), is a multi-component program with several types of interventions involving key actors from various professions (24). Briefly, the method consists of the following components: (1) collaboration among key actors (i.e., owners and staff at training facilities, police officers, and prevention coordinators at county administrative boards and municipalities), (2) training of key actors, (3) policy work and improved enforcement, and (4) certification of training facilities. The importance of policy work for the prevention of substance use in various arenas in the society is supported by previous research (21, 25–27).

The implementation of this method involves several actions. During a standardized training of 7 h, staff at training facilities, police officers, and prevention coordinators, are educated about the police authority's crime prevention work against doping, legislation, and initiatives, sports regulations with Anti-Doping Sweden's controls of gyms, dietary supplements, and techniques for conveying information. Each gym then develops a written policy and action plan and reviews its membership agreements and employment contracts, indicating that they are taking a stand against doping. This method also includes a certification system with a diploma that ensures that certified gyms continuously maintain active doping prevention work. The certification requires that certain standards be met, such as doping training of staff who work at least part-time, an established collaborative relationship with the local police authority and the municipal prevention coordinator, and a specially appointed person responsible for the doping prevention work at the gym. The police carry out inspections and can conduct doping controls among athletes at gyms and training facilities; thus, the relationship between the police and the gym is a central part of 100% PHT. Every other year, the gym is followed-up to ensure that the work continues at a level that corresponds to the requirements of re-certification.

Previous research has shown that the implementation of a method is affected by several hindering or promoting factors (28, 29). Firstly, some factors are linked to the implementation process itself, such as training and coaching of staff in the organization in which the method is to be used. Secondly, implementation is affected by organizational factors, such as attitudes, motivation, and priorities within the organization. Finally, implementation is affected by the external context, i.e., the political orientation, economy, and norms of the surrounding society (28).

The training activities within 100% PHT were put to the test in 2020 and 2021, when restrictions linked to the coronavirus pandemic put a stop to physical (face-to-face) doping training for key actors. To enable training of police officers during this period, STAD developed a digital half-day training on the 100% PHT method. As the police are responsible for ensuring that people comply with doping laws, relevant training is vital (30). Previous research has confirmed that a combination of education, collaboration, and supervision/control (i.e., multi-component intervention) is necessary to reduce availability and counteract substance use (25, 31). STAD's digital training addressed how the police could work with doping prevention and how reasonable suspicion of doping offenses could be built up by gathering evidence. Collaboration with the local media was also highlighted in the training, as well as the exchange of experience among participants from different parts of the country in the form of group discussions.

The purpose of the current study was to identify facilitators and barriers for effective doping prevention at gyms by examining police officers' views on doping as a societal problem, their experiences of doping prevention efforts, and their perceptions on what enables or hinders doping prevention strategies.

## Methods

### Recruitment

A selection of police officers, who had been educated in 100% PHT with experience in doping prevention work, were contacted in November 2021 with a request to participate in an interview study (32). Potential informants were first contacted *via* email informing them of the purpose of the study, how the interviews would be conducted, that participation was voluntary and could be interrupted at any time, and that data was only to be handled by authorized persons and presented in aggregated form so that individual informants could not be identified. After obtaining informed consent, an interview appointment was made, and the informant was provided with a unique code number, which later represented the informant. The interviews were conducted from December 2021 through May 2022. In the end, a total of 15 police officers were included. Background information about professional experience, sex, police region, and role/position for each informant is presented in Table 1. The study was performed in accordance with the Declaration of Helsinki, and the protocol was sent to the Swedish Ethical Review Authority, which judged that ethical permission was not required (registration number. 2019-05156).

**Table 1:** Background information of the informants.

		***N***
Experience as police officer	1–3 years	2
	4–10 years	7
	≥10 years	6
Sex	Female	6
	Male	9
Police region	Bergslagen	1
	Middle	1
	North	2
	Stockholm	2
	South	2
	West	5
	East	2
Role	Investigation leader	1
	Municipality police officer	2
	Intervention police officer	3
	Community police officer (including head of police districts)	8
	Investigating officer	1

### Semi-structured interviews

STAD produced a semi-structured interview guide for assessing the informants' views on doping, how they work against doping, what they perceive as problematic, and what possibilities they apprehend in the prevention of doping (see Appendix 1 in Supplementary material). The guide was based on previous research, outlined in the background section, and on information received from group discussions among police officers who participated in an education on doping prevention. The interviews were carried out by one of the researchers (PK) and were recorded and transcribed verbatim for later analysis. When 15 interviews had been conducted, no or very little new relevant information was expected to be obtained through further interviews, and the interview process was completed.

### Analysis

**Figure 1:**
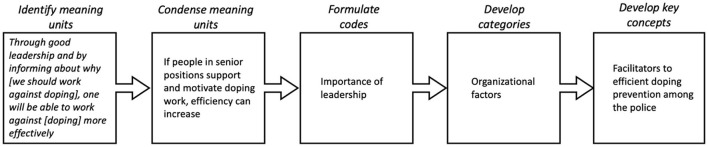
Concept of content analysis.

Qualitative content analysis, inspired by Hsieh and Shannon (33, 34), was used to analyze the interview material. To increase the reliability of the analytical process, a team-based approach (35) was used, which utilized the broad expertise of the research group. Initially, the transcribed interviews were imported into the NVivo 12 program to facilitate structuring and analysis of the material. One of the researchers (PK) repeatedly read through the interviews to identify meaningful units that could be grouped into preliminary codes, categories, and key concepts. The interview questions were indicative in this procedure (33). An example of this analysis process is illustrated in Figure 1.

A coding scheme for the interview material was prepared by PK and presented to another researcher (AS) who used the scheme to independently code five randomly chosen interviews. Subsequently, PK and AS discussed the coding scheme, which did not require modification. The scheme was then presented to the entire research team, who agreed upon the content, consisting of 4 categories and 21 codes, linked to the key concepts' “facilitators” and “barriers,” respectively, as presented in Table 2.

**Table 2:** Coding scheme.

**Categories**	**Doping as a societal problem**	**Interventions at gyms**	**Organizational factors**	**External factors**	**Key concepts**
Codes	Extent	Occurrence	Resource allocation	Political/societal interest	Facilitators/Barriers
	Negative health effects	Preparation	Cooperation	Laws, rules, and practice	
	Violence	Contact with gyms	Knowledge/competence	Police education	
	Skewed beauty ideals	Detection	Motivation	Municipality size	
	Organized crime	Compilation of evidence	Leadership and mandate	Other arenas	
		Media advocacy			

## Results

The informant group consisted of 15 police officers, of whom 2 had worked within the police force for 1–3 years, 7 for 4–10 years, and 6 for 10 years or longer (Table 1). Six informants were women and nine were men. The informants represented all seven police regions in Sweden, however, a disproportionately large number (*n* = 5) represented Police Region West (Table 1). The informants represented a broad variety of professions within the police force, from investigation and intervention police officers to investigation leaders and heads of police districts. They also worked on different levels, including the municipality and police district levels (Table 1). The content analysis resulted in four categories and 21 codes linked to the key facilitators and barriers (Table 2). The results are presented according to the four categories. The numbers in brackets after the quotations represent the informant.

### Doping as a societal problem

The analysis category, “Doping as a societal problem” contains statements concerning the extent of doping, negative health effects, violence, skewed beauty ideals, and organized crime. Most informants regarded doping as a societal problem. Most also observed that doping is relatively common, which is a perception that seems to have been strengthened through participation in the doping education and prevention work in collaboration with gyms.

“It became very clear that this is common. As soon as we stepped into a gym, you could easily identify several persons that were under the influence of doping substances.” (4)

Regular police work also contributed to the perception that doping is rather common, as doping was detected in connection with other crimes, such as drug crimes and assault.

“The problem is that there are a lot of underlying factors with violent crime, gang crime, and so on. We know criminals sell and distribute it [doping substances]…We stumble across doping when we investigate other crimes, first and foremost drug crimes, and in some cases domestic violence.” (14)

Perceiving doping as a societal problem was motivated by its negative health effects, regarding mental health and the fact that doping generates aggressive actions directed at other people, including relatives and even police officers.

“People who use this [doping substance] are at risk of harm and have aggression problems with other people, girlfriends, and partners.” (2)

Another societal problem generated by doping, highlighted by the informants, concerned the doping-produced beauty ideals established through extensive exposure of doped bodies in social media.

“It is mainly younger men, 20–30 years old, who [use steroids] to look big and beautiful.” (6)

This is particularly worrying given that young people can be enticed to achieve an appearance that is impossible to achieve without illegal drugs. These people often do not understand the extent of the long-term effects of doping substances.

“Young people do not understand the consequences of it [doping] when they get older.” (3)

Many of the informants further associated doping with society's problems with organized crime and criminal networks. Selling doping substances can provide income to criminals, which can be used for arms purchases, among other things, according to one of the informants. Additionally, the use of doping among criminals can contribute to increased self-confidence in connection with the use of violence.

“You have it [doping substances] in a gang environment to commit violent crimes…You move your boundaries and get a more grandiose self-image.” (1)

### Interventions at gyms

In the analysis category, “Interventions at gyms,” informants supplied statements concerning the occurrence of interventions, preparatory work, contact with the gym, detection of doping offenses, building a case, and media advocacy. The informants indicated that in some parts of the country, insufficient collaboration between the police and gyms was present. However, they stated that training had improved collaboration, and the police in several police stations had began working with interventions (doping inspections) at gyms.

“At my station, we do not work against doping, we do not visit the gyms, and do not make targeted efforts, but I have been involved in an effort in [X municipality].” (4)

To facilitate the discovery of people in gyms who use doping substances, social media such as Instagram, where many bodybuilders post pictures of themselves is used.

“It's enough that I go out on Instagram and see what is being shown. Many people who appear there use some form of doping. It's hard to look like that otherwise.” (6)

Furthermore, some gym staff contact the police and announce that they suspect that some of their customers use doping substances. Thus, regular contact with the gym and doping inspections by the police is vital for preventive work to be successful.

“If they [gym staff] are not positive to our presence, it will not work in the long run.” (1)

An operation with police officers who enter a gym or stand outside to check suspects is often preceded by civilian police officers themselves training at the facility, as an undercover operation, to identify those who may be suspected of doping offenses. Some police officers have received their own gym cards to facilitate such operations.

“Those who run training facilities want us to come. I have received my own gym membership card, so it has made it easier, of course.” (6)

To gather evidence as a basis for prosecution for doping offenses, a conversation is usually initiated at the gym with the person who is suspected of using doping substances. If reasonable suspicion is established, the person is taken to the station to provide a urine sample, which is then analyzed for illegal substances. Sometimes a house search is also conducted to identify the possession of doping substances. Various forms of dietary supplements may contain illicit substances; however, whether the consumer is aware of this is difficult to determine.

“There are examples of people who bought supplements, and there were illegal preparations in them.” (6)

Most informants do not see dietary supplements as a major problem, but believe that they are problematic if illicit substances are added to the products. According to the informants, whether the evidence collected by the police at the discovery of doping is enough for conviction in court is difficult to know, because the police do not obtain this type of information about individual cases on a regular basis.

“We do not follow up on our own cases. The extent to which it leads to conviction is not really known.” (4)

In interventions made at gyms, contacts with the media often alert society of the issue. The visibility of the issue in the media is seen as an opportunity to strengthen the work against doping. Moreover, to show that the risk of getting caught is real, efforts at the gym are usually combined with education about the efforts *via* media advocacy through the local newspaper or radio channel. However, media advocacy efforts vary.

“We use a lot of social media. So, after the police operation, we announced that we had visited several gyms in [X municipality] to further prevent crime. We want to be visible.” (14)

### Organizational factors

The analysis category, “Organizational factors” contains statements concerning resource allocation, collaboration, knowledge/competence, motivation, leadership, and mandates. The informants' perceptions of resource allocation varied, likely reflecting different conditions among regions and local police areas. A prominent impression is that the issue has come up on the agenda recently and work is now being done to increase efforts.

“It feels like it's an area that has been a bit neglected and that it may not have been seen as a concern, but since it started with initiatives in [X municipality], it has become clear that [doping] is widespread.” (6)

The allocation of resources further depends on other tasks that must be carried out.

“Doping work is part of much else that is done. There are many different things we must work with, of which doping is one, so in everyday work, [efforts against doping] happen if you stumble across it. Otherwise, you must plan such targeted efforts with preparatory work.” (2)

Several local police officers have established collaboration with gyms in the area and receive tips about suspects.

“It was the gym in question that we had received tips about ‘here there may be some [doping]'. So, these people were actually completely unknown to us before we stepped into the gym, but we had a few sessions before when we worked against doping to create contact, when we went around the gyms in [X municipality]. And at some of the gyms we got gym membership cards so we could come and go as we pleased.” (12)

In addition to collaboration with the gyms, the informants suggested other actors that the police could collaborate with. One of the informants emphasized the possibility of cooperating with the Swedish Transport Agency regarding the possibility of revoking driver licenses for those convicted of doping offenses.

“I work with the Swedish Transport Agency, which can withdraw driver licenses. It's probably more efficient than SEK 20,000 [approximately EUR 2,000] in fines.” (5)

Several informants believed that a lack of knowledge regarding the doping issue prevented police and employees from prioritizing doping prevention measures and admitted that their own knowledge had also been limited.

“I think a lot is ignorance about doping… My ignorance has been huge when it comes to doping.” (3)

Lack of knowledge can constitute a barrier in doping prevention. Police officers usually must contact an investigation leader who is often not present at the scene to get permission to test suspects. If the investigating officer is less knowledgeable about doping and how to proceed in case of suspicion of doping, anti-doping work becomes complicated.

“Ignorance among investigation leaders is problematic…We [the investigation leaders] do not have much knowledge when it comes to doping. If you are unsure, you do not dare to make a decision.” (4)

Several informants believed that more training and skills development is needed.

“I think it is important to have some form of basic education in [doping] so that you generate some interest.” (13)

All informants expressed that the training they received on doping was helpful.

“I had some knowledge before [the training] but I think that all training and 100% Pure Hard Training gives tips and tricks on how to proceed and how to start. As long as you have a template to follow that you have after this training, you have a certain security.” (5)

The informants believed that knowledge and motivation are connected and that within the police organization, increased knowledge about the harmful effects of doping in society is needed.

“Then I think that [the police authority] does not see it as such a big problem. There we have to work with attitudes. Because I do not think you see it as a problem. You think it's up to everyone.” (4)

The informants themselves seemed motivated for doping prevention work, which may be because they had recently received a doping education. Several of them were offered to attend the training because they had shown interest in the issue earlier and wanted their colleagues to take part in the training. Many also want to invest more in the doping issue in their local police area.

“I will make a request that we run an effort like this [at the gym]. I hope it will be more regular than it has been and that more people will have the chance to attend the training [on doping].” (3)

A belief in the effects of doping prevention was also prominent, although the idea that doping would disappear completely from society was not perceived as realistic.

“I do not think we will be able to counter doping 100%, but I think we can push it away from the usual gym activities and that is what I think is the most important.” (4)

According to the informants, for doping prevention work to be effective, supportive leadership in the police organization is important.

“Through good leadership and by informing about why [it is important], you will be able to work against [doping] more effectively.” (4)

The distribution of anti-doping work within the police organization is vital to the effectiveness of the work. Several informants who believed that the delegation system may differ for illicit drugs and doping offenses addressed delegation regarding the sampling and arrest of suspects.

“We who have worked for a long time [in the police force] have our own delegation [for] illicit drugs, but we do not have that [for] doping, and that would have made it easier… It's a bit of a hassle for that.” (3)

### External factors

The analysis category, “External factors” includes statements concerning political and societal interests, laws, rules, police education, and municipality size.

Several informants emphasized the importance of the doping issue being on the agenda for effective doping prevention work. One of the informants believed that more research on the association between doping and other crimes could promote an interest in prioritizing the issue.

“In some research studies, the connection between more serious violent crimes and domestic violence and doping is mentioned. And I would really like to see that they did even more thorough research around that. For one of the most important areas we work against is domestic violence.” (4)

“I think that [factors that promote work against doping] are a combination of it being visible in media and that it be discussed within the police authority so that you can target health problems among young people.” (5)

Handling the discovery and collection of evidence for prosecution is governed by laws, rules, and practices. The possibility of gathering evidence increases with the penalty value for various crimes. Some informants reasoned that higher penalties may discourage people from doping.

“Remove the penalty discount!…If the punishment is less severe, you may fall back [into crime]…Many people we talk to say that ‘I can do this because I get nothing for it, I get a fine of about SEK 2,000 [approximately EUR 200]'…There will be no consequences for them.” (13)

However, some informants emphasized the need for more time to carry out the work rather than sharper tools.

“I think we have the tools we need…But it's more that you have enough time, among other things we have to do.” (2)

Some informants felt that contacting the investigation leader during interventions was cumbersome. Additionally, police officers could end up in time-consuming situations if suspects refused to perform urine tests. Some informants believed that taking blood samples to confirm the suspicion of doping offenses would be easier than waiting on urine samples.

“If you could find these substances in the blood, it would have been easier for us, because then we would not be as bound by urine samples.” (10)

Another external circumstance that affects doping prevention work is the extent to which the education at the police academy prepares future police officers. Deficiencies in basic education may have been present.

“It was not much [doping education at the police academy]. I think there was some education, but it was a small part.” (7)

Another external factor, brought up by the informants, is the size of the town the police operate in. In small towns, the police became somewhat local celebrities, hindering undercover operations at gyms.

“They [the residents] know who we are if we go into a gym...One way to get around this may be to swap areas with each other within the region. Within [X region], we have many stations we could switch with.” (3)

According to the informants, people of other occupations should also be trained for a broader reach.

“I believe that we need to educate more of us who work with people in society…Preventive measures really do not just have to be done by the police.” (4)

Other proposals for doping prevention measures that do not fall within the scope of police services include athletes' demands on their sponsors (e.g., athletes should check that their sponsors distance themselves from doping). Additionally, companies that market dietary supplements could declare that they are against doping substances.

“Maybe you can work with communication on websites with dietary supplements…so that they market themselves with pure products and that they are against doping substances.” (4)

Primary care physicians, psychiatrists, and those involved in sports and schools could also work on doping prevention strategies.

“I think you can work with doping substances on many fronts, in schools for example.” (13)

## Discussion

The purpose of the current study was to identify facilitators and barriers of effective doping prevention at gyms by examining police officers' views on doping as a societal problem, their experiences of doping prevention efforts, and their perceptions on what enables or hinders doping prevention work. Interviews were conducted with 15 police officers from all the Swedish police regions representing men and women, various roles in the organization, as well as differences with regard to professional experience. The results are discussed below in relation to previous research, based on how barriers can be removed and facilitators can be promoted to make doping prevention work more efficient.

### Facilitators

Facilitators of effective doping prevention work include the recognition of doping as a societal problem; mobilization of key actors; motivated police management and police officers; adequate resource allocation; cooperation among the police, gyms and other authorities; and competence development for police and other actors. In summary, the informants were aware that doping is a relatively widespread societal issue with health and social consequences. According to the informants, education regarding the prevalence, health consequences, association between doping and domestic violence, skewed beauty ideals, and the link to organized crime can mobilize key actors within and outside the police organization (28, 29). This knowledge motivates leaders and managers to allocate adequate resources, which is an important organizational factor for successful implementation of initiatives (28). The primary recreational doping prevention measure, apart from legislation and control, include efforts to raise knowledge and awareness about the problem, thus in line with the informants' view. Increasing knowledge and awareness can be obtained by educating the target group or stakeholders in different settings, which is further discussed below. Information campaigns in various channels is a way of increasing public awareness of the problem (21). Mass media campaigns are, however, no longer classed as “best practice” and their effects disputed, although experts in the field highlighted the use of athlete role models as potentially effective in national media campaigns (21).

The informants further believed that doping prevention work had been neglected, and awareness of the consequences of doping was not high enough within the police authority or the surrounding society. To increase awareness of the doping problem, the informants proposed further research on the association between doping and other crimes. For example, studies could focus on the prevalence of doping in connection with violent crimes, including domestic violence. Moreover, media advocacy may play an important role in increasing interest in working regionally and locally, which is vital to create external support structures (28, 29).

Establishing and maintaining contact with the gyms is another facilitator for effective doping prevention work. The efforts to create a doping-free environment and limit the availability of doping at gyms are in line with suggestions from experts in the field (21). When police officers sign collaboration agreements with gyms, receive gym membership cards, and visit gyms, they establish a strong relationship with the gyms, promoting the common efforts. In police regions made up of many small towns, different regions can collaborate so that police officers can visit gyms in other regions to avoid being recognized. Furthermore, collaboration among authorities regarding information exchange and sanctions for doping offenses can create a synergistic effect, which is a strategy relying on legislative measures (21). One of the informants highlighted the Swedish Transport Agency's ability to revoke driver licenses in connection with doping offenses, resulting in a more tangible consequence than a fine. Other actors that could generate synergism that were not explicitly addressed by the informants, include Swedish Customs and Postal Suppliers and Anti-Doping Sweden.

Consistent with previous research, the interviews support that education and skills development are vital for streamlining prevention work (28). The informants highlighted educational and motivational aspects of the digital education they had recently completed, while emphasizing the lack of knowledge about doping at the management level within the police organization. They also highlighted the importance of basic training including doping prevention strategies for future officers. In addition, doping training was proposed for investigation leaders. To further increase knowledge about how to work effectively against doping, feedback on whether collected evidence leads to conviction could be considered. One of the informants stated that no systematic feedback is provided regarding whether the discovery of suspects of doping offenses results in sentencing.

The informants also highlighted the potential of doping prevention by involving individuals from other professions, apart from the police, that work with young people, such as primary care physicians, psychiatrists, and those that work in sports and schools, which is in line with previous recommendations (23). Since it is widely recognized that efforts to prevent unwanted behaviors should preferably be carried out during adolescence, the school could be a plausible arena for doping prevention interventions (36). Doping prevention strategies in schools were recently supported by a systematic study of methods for preventing doping among young people (37). However, educational as well as repressive anti-doping has (in the elite sport context) been criticized for being based on a debatable definition of doping as an individual moral fault (38) and there has been difficulties in proving that it changes pupils' or students' attitudes toward doping (39).

To equip different professions for doping prevention work, training efforts on doping may need to be developed. Furthermore, measures were proposed for companies that market dietary supplements to indicate that they support doping-free products. The extent to which legislation on the control of dietary supplements needs to be strengthened is unclear. However, according to the informants, the distinction between doping substances and dietary supplements is often unclear. Considering the connection between doping and domestic violence, social services may also play an important role in doping prevention work.

### Barriers

Barriers to effective doping prevention work include a lack of knowledge about doping, time-consuming processes around the detection and collection of evidence in doping offenses, and competing tasks for police officers. A lack of knowledge has been detected as a key barrier to effective implementation of interventions in previous studies (28, 29). Strengthening basic education and training for police officers and offering further training, both within the police organization and for other professions, is essential.

Furthermore, the informants stated that time-consuming processes around the discovery and collection of evidence in doping offenses constituted a barrier to doping prevention. Waiting for a suspect to provide a urine sample is time-consuming and can sometimes result in suspects being released without providing a sample. The police can only detain people for up to 6 h, providing suspects the opportunity to refuse to give a urine sample. Blood-, saliva-, or breath-tests could remove this barrier (40–42). Some informants also suggested that the absence of mandates for sampling and arrest can be a barrier, especially if the investigation leader (who must be contacted) has a lack of knowledge about doping.

Furthermore, the informants highlighted that a lack of resources and presence of competing tasks is a barrier, which is consistent with a study among key European players in recreational sports (43), as well as general implementation research (28). The key players of European recreational sports stated that the most important barriers to the implementation of prevention measures included a lack of financial and human resources, the absence of collaboration among key players, and a lack of good practice (43). In the present study, collaboration among key actors (i.e., police and gyms) was not a prominent barrier, but rather the opposite. However, this may differ in areas where the police have not attended doping training. Additionally, the lack of good practice was not mentioned by the informants in this study. The impression was instead that the police (informants) believe in the method they are using.

### Strengths and limitations

This study has several strengths that should be highlighted. First, the information was based on interviews with informants that represented a wide range of police regions, years of experience as a police officer, and positions. We assume that all these aspects may influence the perceptions that the informants express. The regions may prioritize the doping prevention work in various ways, experienced police officers can reflect on the changes in Swedish doping policy since the law was implemented, while less experienced police officers may have updated experiences from the police-education that is relevant. Finally, male and female police officers may reflect differently on doping because of own experiences and gender roles (44). Second, a team-based analysis process was used to ensure the reliability of the results. Nevertheless, this study also has weaknesses. First, voluntary recruitment entails a risk of a distorted selection because those that want to participate may differ from those that do not want to participate. Moreover, research based on interviews poses a risk that informants may respond in a way that they believe is expected by the interviewer (45). Finally, the interviewees in our study were recruited from a sample that had taken part in a digital doping training course and had been involved in doping prevention operations at gyms. Thus, our sample consisted of police officers that likely had a higher level of doping-related knowledge compared to the average police officer. This may be viewed both as a study strength and limitation, as these officers may detect facilitators and barriers more easily than those who are not involved in the work. Nevertheless, less experienced police officers may perceive other facilitators or barriers.

## Conclusion

Doping prevention should become more efficient by taking advantage of existing facilitators and removing remaining barriers. This study could guide recommendations linked to the police organization and the surrounding society regarding doping prevention.

## Data availability statement

The data are available from the Centre for Psychiatry Research, a collaboration between the Karolinska Institutet and Region Stockholm, but restrictions apply to their availability, as they were used under ethical permission for the current study, and therefore, are not publicly available. However, data are available from the authors upon reasonable request and with permission from the Centre for Psychiatry Research.

## Ethics statement

The study was performed in accordance with the Declaration of Helsinki, and the protocol was sent to the Swedish Ethical Review Authority, which judged that ethical approval was not required (No. 2019-05156). The patients/participants provided their written informed consent to participate in this study.

## Author contributions

PK: conceptualization, data curation, formal analysis, investigation, project administration, methodology, writing—original draft, and writing—review and editing. AS: data curation, formal analysis, investigation, methodology, validation, writing—original draft, and writing—review and editing. TE: conceptualization, funding acquisition, and writing—review and editing. JG: conceptualization, supervision, funding acquisition, and writing—review and editing. All authors contributed to the article and approved the submitted version.

## Funding

This work was supported by the Public Health Agency of Sweden.

## Conflict of interest

The authors declare that the research was conducted in the absence of any commercial or financial relationships that could be construed as a potential conflict of interest.

## Publisher's note

All claims expressed in this article are solely those of the authors and do not necessarily represent those of their affiliated organizations, or those of the publisher, the editors and the reviewers. Any product that may be evaluated in this article, or claim that may be made by its manufacturer, is not guaranteed or endorsed by the publisher.
