# Supplementary material for: Facilitators and barriers in preventing doping among recreational athletes: A qualitative interview study among police officers
Source: Front Public Health. 2022 Oct 5;10:1017801. doi: 10.3389/fpubh.2022.1017801 (PMC9580715; doi:10.3389/fpubh.2022.1017801)
Supplement: Supplementary file 1 [file Table_1.docx]

**Appendix 1**

***Interview guide***

1) Do you perceive doping and doping-related problems as a societal problem? If, so, in what sense?

- occurrence - user - situations - connection to other crime

2) Can you see any problems with the use of dietary supplements?

- occurrence - user - situations

3) How do you perceive the police's ability to counter doping in society?

- obstacles - facilitating factors - resources - leadership - collaboration with other professions/organizations - legislation

4) What is your experience with doping-related efforts at gyms and training facilities?

- the components of the intervention/s - number of interventions - experience in connection with interventions - obstacles - facilitating factors – cause of intervention - the effect for the individual – the building reasonable suspicion – to get permission from the investigation leader

5) How did you perceive doping-related interventions in gyms and training facilities before yoy took part in the digital doping training for police officers?

- the components of the intervention/s - number of stakes - experience in connection with efforts - obstacles - facilitating factors - reason (what prompted the action(s)?) - the effect for the individual

6) How do you perceive the digital doping training for police officers in terms of preparing officers to carry out such operations?

- knowledge/competence - implementation - quality in the efforts - the consequences of the interventions - frequency
